# Biometric Signals Estimation Using Single Photon Camera and Deep Learning

**DOI:** 10.3390/s20216102

**Published:** 2020-10-27

**Authors:** Marco Paracchini, Marco Marcon, Federica Villa, Franco Zappa, Stefano Tubaro

**Affiliations:** Dipartimento di Informazione, Elettronica e Bioingegneria, Politecnico di Milano, 20133 Milano, Italy; marco.marcon@polimi.it (M.M.); federica.villa@polimi.it (F.V.); franco.zappa@polimi.it (F.Z.); stefano.tubaro@polimi.it (S.T.)

**Keywords:** deep learning, heart rate, remote photoplethysmography, single-photon avalanche diode

## Abstract

The problem of performing remote biomedical measurements using just a video stream of a subject face is called remote photoplethysmography (rPPG). The aim of this work is to propose a novel method able to perform rPPG using single-photon avalanche diode (SPAD) cameras. These are extremely accurate cameras able to detect even a single photon and are already used in many other applications. Moreover, a novel method that mixes deep learning and traditional signal analysis is proposed in order to extract and study the pulse signal. Experimental results show that this system achieves accurate results in the estimation of biomedical information such as heart rate, respiration rate, and tachogram. Lastly, thanks to the adoption of the deep learning segmentation method and dependability checks, this method could be adopted in non-ideal working conditions—for example, in the presence of partial facial occlusions.

## 1. Introduction

Being able to constantly check, in real time and without any contact, the health condition of a person could have a significant impact in many different situations. Possible applications include fitness assessments [1], medical diagnosis [1], and driver monitoring [2]. The act of extracting biomedical information analyzing video capture is called remote photoplethysmography (rPPG) or imaging photoplethysmography (iPPG) [1]. The working principle of this technology is based on the variation of the light intensity reflected from the skin due to the transition of blood in vessels. This generates a signal, called pulse signal, which could be extracted and consequently processed with the aim of estimating the subject’s heart rate and/or other biomedical parameters. Although the pulse signal is different from the electric one generated by the heart activity, due to their own natures, the two are strongly related. Obviously, due to a mechanical delay of approximately 200 ms [3], they are not synchronized, but, on the other hand, they show the same trend since the pressure wave frequency corresponds to the heart beating. Therefore, analyzing the pulse signal is possible to retrieve the heart rate (HR). Further analysis on pulse signal could lead to heart rate variability (HRV) estimation. In particular, the tachogram, which is the time measurement of the time interval between two consecutive R waves [4], could be retrieved from the pulse signal. Moreover, the tachogram’s Fourier transform presents two different main components that are commonly called the low frequency component (LF) and the high frequency component (HF), and the ratio between these two quantities is a measure of the sympathovagal balance, or rather, gives a quantitative information about the activation and functioning of the autonomic nervous system [4]. Finally, the peak of the HF component in a normal subject at rest corresponds to the respiration frequency [4]. For these reasons, performing a spectral analysis of the tachogram could lead to the following information: heart rate, LF/HF balance, and respiration rate. The aim of this work is to propose a method able to estimate the aforementioned biomedical measurements in real time and in a dependable fashion. Moreover, this work explores the possibility of adopting a single-photon avalanche diode (SPAD) array camera instead of a traditional RGB camera, as is done in most publications in this field, e.g., [1,5]. SPAD cameras are able to detect even a single photon [6], they have extremely high frame rate [7], and they have proved their usefulness in a plethora of applications, such as 3D optical ranging (LIDAR) [8], positron emission tomography (PET) [9], and many others. In rPPG applications, SPAD’s high precision can accurately measure the intensity variations of the light reflected by the skin, caused by the blood flowing underneath it. Conversely, the main drawback of using a SPAD sensor is their low spatial resolution due to technical limitation. In order to overcome this problem and use as much spatial information as possible, an ad hoc deep learning-based method is proposed. Finally, since the rPPG estimation of biomedical measures is related to optical signals that could be affected by noise, some dependability evaluation metrics are also proposed. The rest of this work is organized as follows: in Section 2, the rPPG problem and some previous solutions are described; in Section 3, the hardware and software components of the proposed method are described. Following that, in Section 4, a set of experiments are described in order to evaluate the proposed method. Furthermore, in Section 5, the experimental results are reported. Lastly, in Section 6, the conclusions of the this work are discussed.

## 2. Related Work

Contact photoplethysmography (PPG) is a non-invasive and simple technique introduced in the 1930s [10]. Using this approach, microvascular blood volume changes in tissues are measured using light [11]. These periodic changes are related to the heart activity [11]. More recent publications, i.e., in 2008 [12], show that PPG could be performed remotely (i.e., rPPG) using ambient light as the optical source. Many other rPPG focused studies were published shortly after [5,13,14,15,16,17]. Some surveys on the state of the art of this field could be found in [1,18,19,20]. While machine learning techniques are widely used in contact PPG applications [21], recent works [22,23,24] explored the opportunity of also using deep learning methods in remote PPG applications. All these works completely substitute the classical signal processing techniques with deep learning ones using an end-to-end network, as in [22,24], or using two consecutive neural networks, as in [23]. On one hand, the use of an end-to-end deep learning model has proved to achieve state-of-the-art results on many computer vision tasks such as image segmentation, object detection, and many others. On the other hand, this kind of method required a massive amount of training data in order to learn how to extract heart-related information directly from video frames without incorporating any prior domain knowledge. This make the performance of this kind of method tightly linked to the training data set and potentially unable to generalize in different setting conditions. Moreover, the complete substitution of classical signal processing techniques developed using a solid theoretical background (signal filtering, Fourier transform, etc.) with data driven ones could lead to non-optimal solutions. To the best of our knowledge, no prior work has been done in trying to combine traditional and deep learning-based signal processing in this field. Lastly, in all the considered studies, the cameras used are traditional RGB cameras. The main aim of this study is to validate the effectiveness of performing rPPG using an SPAD camera, in particular in low illumination conditions, coupled with a deep learning technique in order to compensate for low spatial resolution of single-photon cameras. Adopting a SPAD camera could also be beneficial in the use of the proposed rPPG system in uncontrolled environments in which there could be sudden light variations (for example, if this technology is used in order to monitor a driver, this could happen in tunnel or in presence of car light reflexes). In this kind of scenario, the best strategy in order to remove this high frequency noise is oversampling, and SPAD cameras are the best in this field.

## 3. System Overview

**Figure 1:**
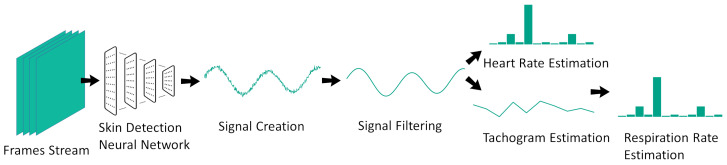
The proposed remote photoplethysmography (rPPG) method. The frame stream coming from the single-photon avalanche diode (SPAD) camera is first analyzed with a neural network that generates a signal further processed with classical techniques.

As with many others rPPG applications [5], the one proposed here could also be split into two successive steps: signal extraction and signal analysis. A scheme depicting the complete work flow is reported in Figure 1.

### 3.1. Signal Acquisition

One of the main contributions of this work is the use of SPAD cameras instead of traditional ones as the acquisition device. The ability of SPAD cameras is to work in dark environments in which the light signal detected by standard CCD or CMOS cameras could be very low; this is the main advantage of the proposed system. This is made possible since these cameras make use of SPAD sensors instead of conventional pixel ones that convert the arrival light in electric charge proportionally. On the other hand, the main drawback of using a SPAD camera is that the SPAD sensors technology do not allow SPAD cameras to have high spatial resolution. The one used in this work, which is a SPC3 camera developed by MPD http://www.micro-photon-devices.com/Products/SPAD-by-Technology/Standard-CMOS/SPC3, has a resolution of just 64 × 32 pixels. The use of a deep learning-based method in the signal extraction step, described in Section 3.2, is a critical component introduced to overcome the low spatial resolution. The SPAD frame rate is set to 100 Hz.

### 3.2. Signal Extraction

The signal extraction phase is composed of two components (facial skin detection and signal creation) which are illustrated in the next paragraphs.

#### 3.2.1. Skin Detection

**Figure 2:**
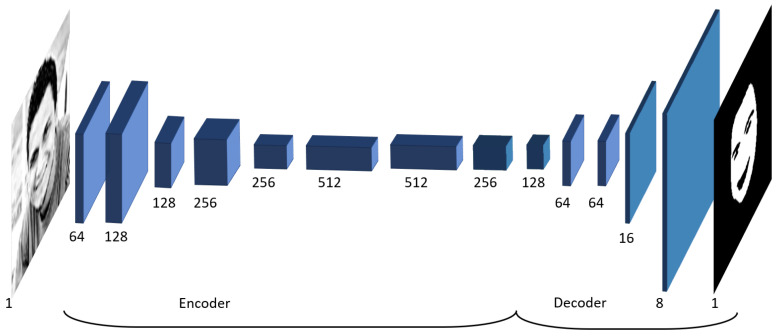
Facial skin network topology. For each layer, the number of filters is reported.

The majority of rPPG applications [5] make use of face detection methods in order to localize specific regions of the subject face where the pulse signal is extracted. In the proposed work, a convolutional neural network is used instead. The chosen network has a U-shape [25], takes a low resolution grayscale image as an input (exactly the same kind of frames produced by the SPAD camera), and produces as an output a single channel image, with values between zero and one. In particular, these represent for each pixel the estimated probability of depicting a skin region. This method is robust to occlusions and, by considering all the visible facial skin surface, overcomes the problem of selecting a restricted skin region (that could be easy occluded) a priori. As reported in Figure 2, the first part of the network (i.e., encoder) is composed of eight consecutive convolutional layers, using 3 × 3 kernels, coupled with ReLu nonlinear activation functions. In addition to that, three max pooling layers are adopted in order to obtain in the last encoding layer a tensor with one-eighth of the original input spatial dimension. Conversely, the second part (i.e., decoder) is constituted by six layers using 3 × 3 kernels and ReLu activations, with the exception of the last one, which uses a sigmoid function to obtain output values in the desired range, i.e., [0, 1]. These are coupled with upconvolutional layers introduced in order to increase back the spatial dimension to the input one. Since the facial skin detection problem is very specific, unfortunately, only a limited amount of data is available for this specific problem. For this reason, a transfer learning approach has been adopted in the training phase. In particular, the proposed skin detection network architecture was chosen in order to have most of the layers in common with a convolutional neural network proposed to solve the grayscale images colorization problem [26]. These apparently different problems are, in reality, tightly linked as a colorization method; in order to work on face images, it must (implicitly) solve the skin detection problem, since it needs this information in order to color in a correct way pixels depicting skin regions. On the other hand, since the skin detection problem is only a small sub-task in respect to the colorization one, the proposed network was significantly simplified. In order to train the skin detection exploiting the colorization network knowledge, a two-step transfer learning strategy was adopted. Firstly, the colorization method was trained on a large data set of unlabeled face images. This was done in order to drive the preliminary method into the specific domain of face image analysis. The skin detection network was subsequently trained starting from the colorization network weights and minimizing the following asymmetric loss function.

(1) $$E\left(\hat{y},y\right)=\sum_{ij}{\left(y_{ij}-{\hat{y}}_{ij}\right)}^{2}\left(\alpha \cdot y_{ij}+\left(1-\alpha \right)\left(1-y_{ij}\right)\right)$$

where *y* and $\hat{y}$ are the network output and ground-truth skin masks, respectively, and α∈[0,1] is a parameter introduced in order to make *E* asymmetric. We choose a value for α smaller than 0.5, e.g., 0.4, in order to penalize false positive errors (i.e., ${\hat{y}}_{ij}=1$ with $y_{ij}=0$). The skin masks training data set, containing more than 6000 labeled face images, was ad hoc created for this task. The complete data set is available for download at the link: https://github.com/marcobrando/Deep-Skin-Detection-on-Low-Resolution-Grayscale-Images. Further detail on the chosen architecture and training procedure are described in [27]. Although the SPAD acquisition frame rate is set to 100 Hz, the deep learning skin detection method is executed at 10 Hz on key frames obtained by averaging 10 consecutive frames. This is done mainly for computational reasons and to reduce acquisition noise (further detail on SPAD sensors noise can be found in [6]).

#### 3.2.2. Signal Preprocessing

For each frame acquired, once the relative skin detection output is available, a binary skin mask is obtained by comparing the skin detection output to a fixed threshold, optimized during training. The raw pulse signal is then obtained by averaging the intensity value of all the pixels inside the binary skin mask. The values respectively below and above the 10 and 90 percentiles are removed before computing the average in order to exclude possible outlier values that could be caused by errors in the skin detection step. Moreover, in order to remove considerable jumps from the preprocessed pulse signal due to the skin mask variations, an offset value is removed before concatenating the average value to the pulse signal. Furthermore, the maximum signal buffer size has been set to 6000, which corresponds to one minute of observations sampled at 100 Hz. This has been done for the sake of increasing the estimations’ stability by obtaining them on a sufficiently long period of time without excessively increasing their latency.

### 3.3. Signal Processing

After the signal has been extracted, the signal processing step is performed in order to extract relevant information from the obtained pulse signal.

#### 3.3.1. Filtering

A bandpass Butterworth filter is applied to the signal obtained as described in Section 3.2. The filter bandwidth is between 0.4 Hz and 4 Hz, which is equivalent to 24 bpm and 240 bpm. In particular, the chosen filter has real zeros in −1 and 1 and real poles in 0.824 and 0.966. This is mainly done in order to cut out any other signal having a frequency very distant from a possible HR.

#### 3.3.2. Average Heart Rate Estimation

The next step is the estimation of heart rate. In order to achieve this, two consecutive steps are applied. Firstly, a fast Fourier transform (FFT) is applied on the filtered signal obtained as described in Section 3.3.1. Lastly, the peak of the signal in the frequency domain is selected, thus estimating the subject average heart rate.

#### 3.3.3. Tachogram Estimation

In order to achieve a good tachogram estimation, all the heart beat peaks in the considered time interval must be precisely detected. For this reason, the local maxima of the filtered signal obtained as described in Section 3.3.1 are detected using two different criteria. The first one is a threshold related to the minimal temporal distance between consecutive maxima while the second criteria is related to the amplitude of the maxima, following a non-maximal suppression approach. After this phase, the information obtained about the average RR time interval is used in order to modify the temporal threshold in order to search for missing pulse peaks by forcing the searching function to find a maximum inside each temporal window around the calculated average inter-beat interval. As described in [28], the obtained tachogram is re-sampled in order to recover an evenly sampled temporal series.

#### 3.3.4. LF/HF Estimation

LF and HF components were obtained by applying FFT to the tachogram obtained as described in Section 3.3.3. Following the definition, for the two tachograms, LF is computed as the area under the power spectral density (PSD) curve between 0.04 Hz and 0.15 Hz, while HF is calculated as the area from 0.15 Hz to 0.4 Hz.

#### 3.3.5. Respiration Rate Estimation

After computing the tachogram PSD, the respiration rate estimation was calculated as the frequency corresponding to the maximum value in the HF range (i.e., 0.15 Hz to 0.4 Hz interval) of the PSD of the tachogram.

### 3.4. Dependability Processing

Given that rPPG is an optical method, it could be affected by optical alterations. In particular, some scenarios could occur in which the pulse signal could be masked by much stronger noise due to many different sources. The two scenarios that we identified are the presence of subject head periodic movements and background pulsating light.

#### 3.4.1. Periodic Head Movements

**Figure 3:**
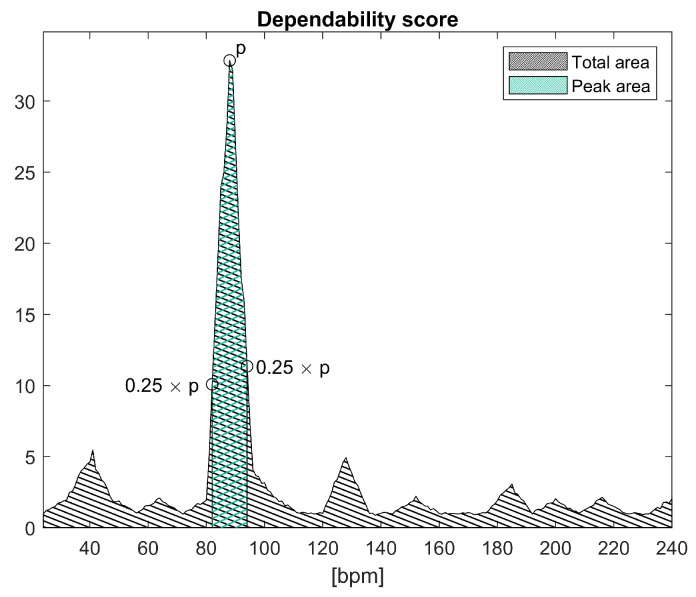
Dependability score definition. Both periodic head movement and pulsating light scores are defined as the ratio between area under peak (green) and total area (black).

The estimation of the main pulse signal frequency could be affected by periodic head movements. In particular, such movements are the HR frequency band and could mask the true HR frequency altering the rPPG HR estimation. For this reason, a visual-based method able to detect periodic head movements has been developed. The first step of this method is to keep track of the head position for each analyzed frame. In order to do this, for each key frame, the central skin mask point is tracked, averaging the coordinates of the skin mask itself. Although this simple method could introduce some errors, particularly in the case of face rotation, it is suitable for a real-time implementation due to its low computational cost. Once the two (vertical and horizontal coordinates) time-varying variables related to the pixel position of the face have been estimated, a principal component analysis (PCA) is used in order to combine this information into a single signal. In particular, the PCA is used to find the principal axes that compose the movement and the coordinates are projected to the principal component. This process creates a 1D time-varying signal on which FFT is applied in order to estimate its main frequency. Its signal power spectrum is then used in order to estimate a score defined as the percentage of the area below the peak in respect to the total area below the power spectrum graph (Figure 3). In particular, the area under the peak is defined as the area below the graph between the interval defined by the two points respectively on the left and right of the peak in which the curve value reach 25% of the peak one. Ideally, in the presence of periodic head movement, a single peak would be visible in the power spectrum, so the score would be very close to 100% (its maximum value). On the other hand, if the peak would not be clearly visible in the power spectrum (due to noise), the score would be much lower. The periodic head movement is then detected using the aforementioned movement-related score—in particular, checking if the score value is greater than a fixed threshold, optimized during a training procedure. In this way, periodic head movements could be detected.

#### 3.4.2. Pulsating Light

Another possible situation in which the rPPG method could lead to incorrect results is in the presence of strong pulsating ambient light in the same typical band of the HR. This situation could occur, for example, while driving in a tunnel; in this case, the intensity of the light that illuminates the driver’s face varies in time in respect to the distance of the closest lamp. In this situation, the ambient light fluctuations would add up to the ones related to the heart activity in the observed pulse signal and, if the first ones are strong enough, would mask the HR-related information. As for the periodic movement detection, an auxiliary signal is needed in order to detect this situation. In particular, an additional environmental intensity signal is extracted, averaging the value of background pixels. These are defined as the pixels of the image outside the detected skin mask. The background signal power spectrum is then extracted via FFT. In this case, a score is defined as the area below the main peak divided by the total area below the power spectrum graph (i.e., the total power). The score obtained is then used in order to detected pulsating ambient light comparing its value to a fixed threshold, optimized during a training procedure.

## 4. Method

In this section, four different experiments are described in order to try to answer to the following questions: Is it possible to perform rPPG using a SPAD camera? Which is the best light wavelength in order to perform it? How do SPADs compare to traditional RGB cameras in this task? What are the major improvement of utilizing a deep learning skin detection method? How effective are the dependability checks introduced in Section 3.4? Moreover, in order to answer the aforementioned questions, some common evaluation metrics are described in Section 4.5.

### 4.1. Experiment 1—Wavelength Selection

The first experiment tackles the problem of determining which illuminant wavelength is optimal in performing rPPG using the SPAD camera. For the sake of finding the optimal optical wavelength, different optical filters were used in order to find out which wavelength results in containing the greatest amount of information related to pulse wave. In particular, physical optical filters were put in front of the lens so just the selected light component would be captured by the sensor. Ten different optical filters starting from 400 nm, blue light, up to 850 nm, and infrared light, with 50 nm steps, were used for this comparison. This wavelength range was chosen in order to match the spectral range of the SPAD camera. Each one of these optical filters implements a bandpass filter centered around each specific wavelength with a full width at half maximum (FWHM) of 40 nm. In this first setup, five subjects had been recorded using all filters; each cardiac activity was also monitored using a portable ECG recorder (Faros 180 http://ecg.biomation.com/faros.htm). Recording sessions were always taken in resting conditions, i.e., subjects seated and facing the camera while avoiding head movement, and each acquisition lasted for 10 min. In order to obtain a wide spectrum in the light source, different kinds of illuminants were considered and tested; finally, an incandescent lamp was chosen. Acquisition frequencies were set at 100 Hz and 250 Hz for the SPAD camera and the Faros ECG, respectively.

### 4.2. Experiment 2—SPAD and RGB Cameras Comparison

After selecting the best illumination wavelength, another experiment was set up in order to compare the accuracy in rPPG applications of the SPAD camera versus a traditional RGB camera. To achieve this goal, a Basler GigE RGB camera was employed. In particular, the model of the chosen camera is acA1920-48gc https://www.baslerweb.com/en/products/cameras/area-scan-cameras/ace/aca1920-40gc/, which is a microcamera that can reach up to 50 fps with global shutter and a resolution of 1920 × 1200 pixels with a CMOS sensor. Sensor dimensions are 9.2 mm × 5.8 mm with pixel size of 4.8 μm × 4.8 μm. In order to perform the comparison between RGB and SPAD cameras, three subjects had been recorded using both cameras and the Faros portable ECG for 10 min each at resting conditions. SPAD and RGB cameras were put side by side at an approximate distance of 50 cm from the subject’s face. Lenses were chosen in order to record the entirety of each subject face from both cameras. The 550 nm optical filter was mounted on the SPAD camera since it produces the best results in the wavelength selection experiment, as will be described in Section 5.1. The same incandescent lamp as the former experiment was also used in this case. Acquisition frequencies were set at 100 Hz, 50 Hz, and 250 Hz for the SPAD and RGB cameras and the Faros ECG, respectively. For each acquisition, the two cameras were synchronized via software.

### 4.3. Experiment 3—Deep Learning-Based Signal Extraction

In order to test the advantage of using a deep learning skin detection algorithm instead of a classical face detection method, a specific experiment has been performed. In particular, the heart rate estimation obtained with the method described in Section 3.3.1 has been compared to the one obtained with a classical rPPG approach [14]. In classic rPPG, an optimal face region (usually the forehead) is detected by applying a fixed proportion to a bounding box obtained with classical face detection methods (e.g., [29]). In order to test the differences between the two methods, two signals have been extracted and analyzed with the same processing described in Section 3.3. In particular, one signal has been obtained using the proposed skin detection algorithm while the other one was extracted from the forehead region obtained applying fixed proportion to the face bounding box obtained with the method in [29]. Moreover, an additional signal has been extracted by removing from the skin binary mask the forehead region obtained using the same face detection method considered above (i.e., [29]). This was done in order to test the scenario in which the forehead region is unavailable—for example, in case of occlusion due to hair presence of wearable objects. Two sequences with two different subjects (one male and one female) were recorded while driving in a car simulator. The SPAD camera, equipped with a 850 nm optical filter, was mounted approximately at 50 cm from the subject’s face and an active infrared illumination was used. The grand truth heart rate values was obtained with the Faros ECG device.

### 4.4. Experiment 4—Dependability Checks Evaluation

The dependability checks described in Section 3.4 have been evaluated experimentally in two different set of acquisitions. For each one of the two checks, two sequences with two subjects (one male and one female) were recorded while using the same driving simulator described above. In this case as well, the SPAD camera, equipped with the 850 nm optical filter, was mounted approximately at 50 cm from the subject’s face. In the two sequences recorded in order to test the ambient pulsating light, an incandescent lamp was used. This external light source was modulated at a frequency of 60 Hz and was turned on with a random delay from the record starting and the delay in the detection time (using the algorithm described in Section 3.4) was recorded. On the other hand, in order to test the periodic head movement detection, the external light source was not used, and instead, the subject was asked to start moving periodically their head left to right at a fixed frequency of approximately 1 Hz. In this case as well, the detection time of the periodic head movement was recorded.

### 4.5. Evaluation Metrics

In order to quantitatively evaluate the results of experiments described in the previous subsections, five different parameters are introduced and considered. For each one of them, a brief description and definition is given in the following paragraphs.

**Single beat detection** The first parameter considered is the accuracy in the single beat detection, which represents the capability of the acquired signal to produce an average wave shape recognizable as an heart beat (qualitative evaluation) and with a small standard deviation (quantitative evaluation). Exploiting a reference groundthrout ECG tack, all the time positions of the QRS complexes were determined using the Pan–Tompkins algorithm [30]. A segmentation of the pulse signal is then obtained in which each element represents a signal portion relative to a specific heart beat. Therefore, after resampling each segmented heart beat wave in order to have the same amount of sampling points, each pulse wave was normalized using $L^{2}$ norm.

**Heart rate** The second metric chosen is the computed HR estimation. The average HR error is defined as the absolute difference between the average HR estimation obtained from the SPAD signal and the one obtained from the ECG trace (considered as ground truth).

**Tachogram** The third considered figure of merit is the tachogram estimation error. The tachogram estimation error is calculated using the root–mean–squared error (RMSE) between the tachogram estimated with the SPAD signal and the one obtained with the ECG groundthruth. The processing steps performed for the tachogram estimation are described in Section 3.3.3.

**LF/HF** The spectrum of the thacogram presents two different main components that are commonly called the low frequency component (LF) and the high frequency component (HF). These two components are defined as the integral of the spectrum in the following ranges of frequency: LF from 0.04 to 0.15 Hz, while HF from 0.15 to 0.4 Hz. The fourth considered metric is the LF/HF estimation error, and it is calculated as the percent error between the LF/HF ratio obtained starting from SPAD rPPG signal and the ECG track, respectively. **Respiration Rate** The HF component of the tachogram is also known as the respiratory band, and, in particular, the peak of the HF component in a normal subject at rest condition corresponds to the respiration frequency [31]. The last metric introduced in order to chose the best illumination wavelength is the respiration rate estimation error, calculated as the absolute error between the respiration rate obtained with the SPAD signal and the one from the ECG expressed as breaths per minute.

## 5. Evaluation Results

In this section, results obtained from performing the experiments described in Section 4 are reported.

### 5.1. Experiment 1—Wavelength Selection

**Figure 4:**
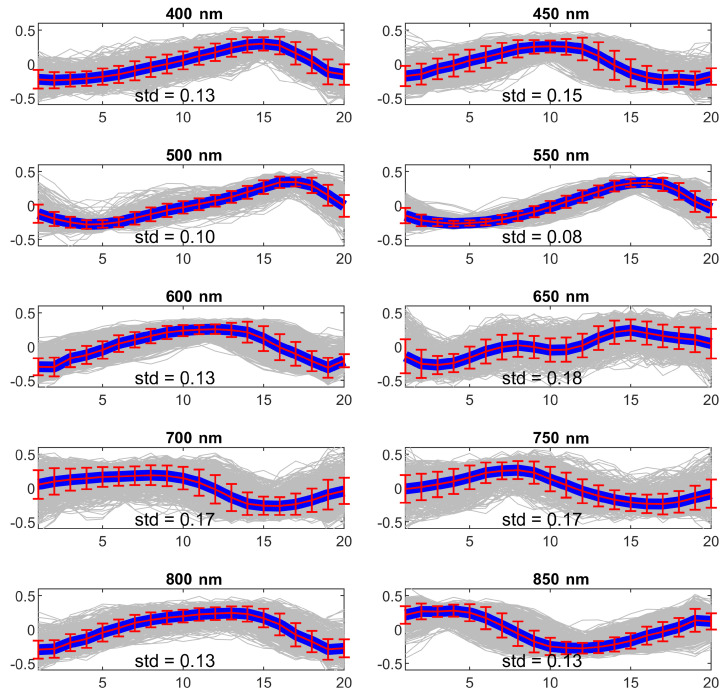
Average beat shape for subject 1 for each one of the wavelengths. Each beat shape is reported in gray and the blue lines represent the average. The red intervals represent the standard deviation for each sampling point. Values on the *y*-axes are normalized amplitudes.

**Heart beat estimation** In Figure 4, the obtained average beat shapes for one of the subject is reported for each filter at different wavelengths. In particular, each beat shape is reported in gray and the blue line represents the point-wise median. The red intervals represent the standard deviation for each sampling point. As can be observed for some wavelength the beat shape is not recognizable (e.g., 650 nm) while for other the pulse wave is clearly visible (500 nm, 550 nm). The standard deviations for all the subjects and all the wavelengths are reported in Table 1. As can be observed, the 550 nm wavelength generally produces more precise results.

**Heart Rate**Table 2 reports the result in the average heart rate estimation. It should be noted that, in some tables, reported N.A. values mean that the original signal carried so little information about the pulse wave that the analysis could not be performed. As can be observed, estimations obtained using 500 nm and 550 nm achieve the best results since the mean absolute error is less then 2 bpms for both wavelengths.

**Figure 5:**
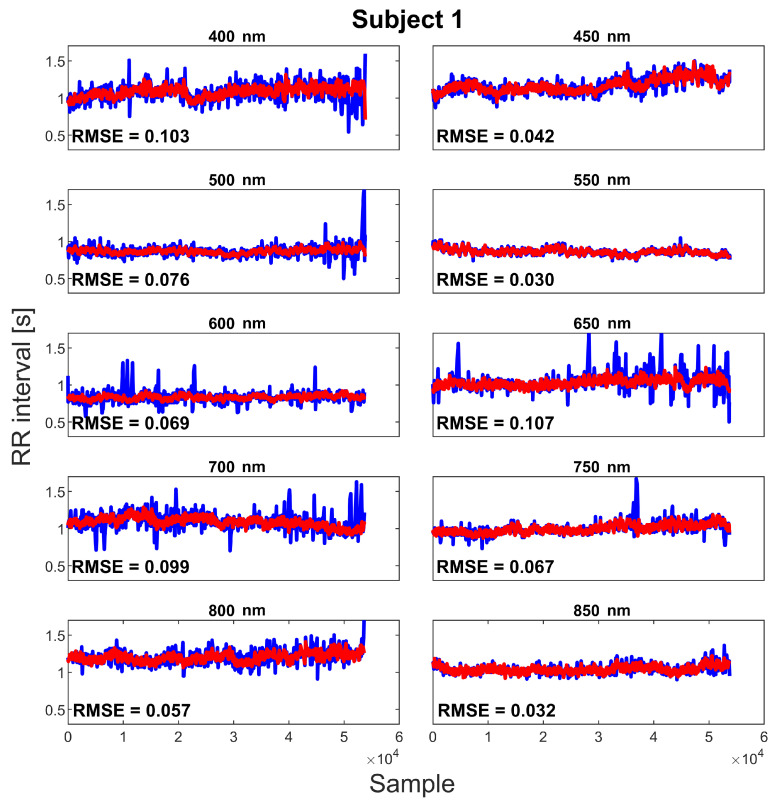
Example of tachogram result. Blue: tachogram extracted by pulse wave; red: tachogram calculated by ECG track.

**Tachogram** In Figure 5, the tachogram extracted from the SPAD camera (blue lines) and from the portable ECG device (red lines) are reported for all the acquisitions of one of the experiment subjects. As can be observed, all the estimated SPAD tachograms correctly have the ground truth line as the mean value. In particular, the one obtained with the filter at 550 nm wavelength is the one with the lowest fluctuations. In Table 3, the complete RMSEs between the estimated curves and the ground truth ones are reported. As can be observed, the lowest errors are reached while using the 550 nm wavelength filter.

**LF/HF** Furthermore, in Table 4, the HF/LF ratio percent errors are reported. As can be observed from performing rPPG using the SPAD camera and the tachogram estimation techniques described in Section 3.3.3, one could retrieve some information on a relatively hard task as remotely estimating the sympathovagal balance. In particular, the best results are achieved at the 550 nm wavelength with a average RMSE of 0.8, which is a state of the art result as reported in [32].

**Respiration Rate** Lastly, in Table 5, the respiration rate errors are reported. As can be observed, the respiration rate could be estimated with a high accuracy using all the different wavelengths and reaching the best results while using the 550 nm optical filter.

### 5.2. Experiment 2—SPAD and RGB Cameras Comparison

**Heart Rate** Heart rate estimation results are shown in Table 6. The table shows that the developed setup and signal processing allow a high accuracy in the determination of the heart rate, showing an average error lower than 0.2 bpm.

**Tachogram** The deviations were calculated as root–mean–squared error between the tachogram from the SPAD and the one from ECG, and between the tachogram of the RGB camera and the ECG.

The deviations calculated over the entire tachograms are reported in Table 7.

From that table, for two subjects (Sbj1 and Sbj2), results are equivalent in terms of accuracy, while for the third subject, the SPAD camera returns better results because of a beat missing in the RGB tachogram estimation.

### 5.3. Experiment 3—Deep Learning-Based Signal Extraction

Results are reported in Table 8. As we can observe, the use of the proposed skin detection method performs as well as using classical face detection methods. In addition to that, the proposed method has the benefit of also working in situations in which the forehead skin intensity is not available, as can be observed from the last row of the table.

### 5.4. Experiment 4—Dependability Checks

In all of the four tested sequences, the optical noise injected was correctly detected. In particular, the delay detection for pulsating light has been of 13 seconds and 15.5 seconds for periodic head movements. These delays were expected due to the one-minute signal window used and described in Section 3.2.

## 6. Discussion and Conclusions

This work introduces a new rPPG method that exploits SPAD cameras instead of traditional ones and mixes deep learning and traditional signal processing techniques. The working principle and reason behind the use of SPAD cameras had been discussed in Section 3.1. In particular, SPAD cameras, thanks to their high frame rate, are more suitable to many uncontrolled light scenarios in which the system could adapt to sudden illumination changes.

An extensive study was conducted (experimental setup described in Section 4.1) in order to compare the SPAD rPPG performance using light with different wavelengths. As can be observed from results reported in Section 5.1, 550 nm light (i.e., green light) is able to achieve better results. Many parameters influence this result; in particular, the most significant are light penetration depth in the tissues [33], absorption coefficient of the oxygenated hemoglobin [34], SPAD efficiency [6], and illumination power. Light below 500 nm is mostly reflected by stratum corneum, which is the most external skin layer, which, being unreached by blood, does not contain any information on pulse wave. Concerning light between 600 nm and 750 nm, the absorptivity of oxygenated hemoglobin is very low, thus reducing the modulation in rPPG signal. Therefore, only wavelengths between 500 nm and 600 nm and between 750 nm and 900 nm are able to carry a useful signal. As a matter of fact, as shown from the results reported in Section 4.1, the best performance is achieved using 550 nm light, but reasonable results are also achieved using near-infrared light (750 nm to 850 nm). These are promising results since many scenarios could be imagined in which the use of non-visible light could be preferred (e.g., in the automotive field, an rPPG system could be used in order to monitor the health state of the driver).

The second experiment (described in Section 4.2) was conducted in order to compare the rPPG SPAD-based performance with the one obtainable using traditional RGB cameras. As can be observed in a normal light scenario, as reported in Section 5.2, SPAD cameras are able to achieve comparable results in respect to RGB cameras in heart rate estimation and slightly superior accuracy in estimation of the tachogram.

In Section 3.2.1, the adoption of a deep learning-based method for facial skin segmentation has been illustrated. In particular, the main motivation for utilizing a segmentation method was to be able to use all the possible pixel surface related to the heart activity. As a matter of fact, using a traditional forehead region adopted in many rPPG systems [14], given the very low spatial resolution of SPAD cameras, would result in selecting very few pixels for the pulse signal estimation. The results reported in Section 5.3 show a slight increment in heart rate estimation accuracy while using the deep learning skin segmentation method instead of the forehead region obtained with traditional computer vision techniques. More importantly, this experiment highlights how the rest of the skin region detected by the deep learning method, excluding the forehead region, still carries pulse information, and this method could achieve good quality results even in the presence of occlusions (e.g., caused by wearable objects or hair) that could make the forehead region unavailable. Finally, the proposed dependability checks proved to be effective at detecting problematic situations in which the pulse signal could be masked and mistaken by other optical related signals.

## Figures and Tables

**Table 1:** Standard deviations for the single beats detection for each acquisition. Bold values represent best results.

Wavelength (nm)										
	**400**	**450**	**500**	**550**	**600**	**650**	**700**	**750**	**800**	**850**
**Sbj 1**	0.14	0.15	0.10	0.08	0.13	0.18	0.17	0.17	0.13	0.13
**Sbj 2**	0.14	0.12	0.12	0.12	0.14	0.16	0.16	0.15	0.14	0.12
**Sbj 3**	0.14	0.14	0.11	0.12	0.12	0.20	0.21	0.22	0.16	0.19
**Sbj 4**	0.15	0.12	0.13	0.12	0.15	0.21	0.20	0.20	0.14	0.13
**Sbj 5**	0.15	0.12	0.12	0.09	0.12	0.15	0.16	0.14	0.11	0.10
**Avg.**	0.14	0.13	0.12	**0.11**	0.13	0.18	0.18	0.18	0.14	0.13

**Table 2:** Errors calculated as absolute differences between heart rate obtained from the electrocardiogram (ECG) track and SPAD pulse signals. Bold values represent best results.

Wavelength (nm)										
[bpm]	400	450	500	550	600	650	700	750	800	850
**Sbj 1**	0	2	4	1	3	7	4	2	1	0
**Sbj 2**	27	41	0	0	0	9	32	22	50	24
**Sbj 3**	18	1	3	2	14	15	12	17	9	18
**Sbj 4**	0	0	0	1	2	12	N.A.	6	1	0
**Sbj 5**	4	6	3	0	3	1	N.A.	1	0	0
**Avg.**	9.8	10.0	2.0	**0.8**	4.4	8.8	16.0	9.6	12.2	8.4

**Table 3:** Errors calculated as mean square error (MSE) between tachogram obtained from ECG track and SPAD pulse signals. Bold values represent best results.

Wavelength (nm)										
[s]	400	450	500	550	600	650	700	750	800	850
**Sbj 1**	0.10	0.04	0.08	0.03	0.07	0.11	0.10	0.07	0.06	0.03
**Sbj 2**	0.05	0.07	0.03	0.07	0.23	0.22	0.23	0.15	0.09	0.06
**Sbj 3**	0.09	0.07	0.06	0.03	0.08	0.17	0.17	0.15	0.10	0.28
**Sbj 4**	0.07	0.05	0.05	0.03	0.07	0.21	N.A.	0.18	0.11	0.07
**Sbj 5**	0.12	0.10	0.14	0.02	0.08	0.30	N.A.	0.12	0.05	0.05
**Avg.**	0.09	0.07	0.07	**0.04**	0.11	0.20	0.17	0.13	0.08	0.10

**Table 4:** High frequency (HF)/low frequency (LF) root–mean–squared error (RMSE) between the SPAD estimation and the ECG ground truth one. Bold values represent best results.

Wavelength (nm)										
	400	450	500	550	600	650	700	750	800	850
**Sbj 1**	1.6	1.5	1.1	1.3	2.1	1.6	1.7	1.2	1.3	1.5
**Sbj 2**	0.3	0.4	1.2	0.5	0.9	0.9	0.5	0.6	0.3	0.3
**Sbj 3**	1.0	1.7	1.4	1.3	1.8	4.2	1.2	2.8	2.5	3.3
**Sbj 4**	1.3	1.2	1.8	0.8	1.1	1.9	N.A.	1.5	1.8	1.7
**Sbj 5**	1.2	0.8	2.5	0.1	0.5	1.4	N.A.	0.8	0.6	0.6
**Avg.**	1.1	1.1	1.6	**0.8**	1.3	2.0	1.1	1.4	1.3	1.5

**Table 5:** Respiration rate errors between the SPAD estimation and the ECG ground truth one. Bold values represent best results.

Wavelength (nm)										
[bpm]	400	450	500	550	600	650	700	750	800	850
**Sbj 1**	0.4	0.3	0.4	0.2	0.4	0.7	0.5	0.4	0.3	0.3
**Sbj 2**	0.4	0.2	0.4	0.0	0.2	0.3	0.4	0.1	0.3	0.1
**Sbj 3**	0.3	0.5	0.1	0.1	0.7	0.4	0.7	0.8	0.6	0.5
**Sbj 4**	0.3	0.1	0.3	0.5	0.4	0.5	N.A.	0.6	0.6	0.4
**Sbj 5**	0.4	0.7	0.4	0.3	0.2	0.7	N.A.	0.2	0.6	1.1
**Avg.**	0.36	0.36	0.32	**0.22**	0.38	0.52	0.53	0.42	0.48	0.48

**Table 6:** Average errors in determination of heart rate in one-minute windows.

Error [bpm]	SPAD	RGB
**Sbj 1**	0.0	0.0
**Sbj 2**	0.2	0.2
**Sbj 3**	0.2	0.2
**Avg.**	0.1	0.1

**Table 7:** Mean square error between tachogram extracted from cameras and ECG. Bold values represent best results.

RMSE (ms)	SPAD	RGB
**Sbj 1**	2	2
**Sbj 2**	0.8	0.7
**Sbj 3**	0.6	6
**Avg.**	**1.1**	2.9

**Table 8:** Comparison of heart rate estimation between signal extracted with deep learning-based facial skin detection (**Skin**) versus classical face detection method (**Foreh.**).

	Skin		Foreh.		Skin w/o Foreh.	
(bpm)	RMSE	std	RMSE	std	RMSE	std
**Sbj 1**	2	1.4	2	1.4	2	1.4
**Sbj 2**	1.4	0	1.4	1.4	1.4	0
**Avg.**	1.7	0.7	1.7	1.4	1.7	0.7
